# Leucokinins: Multifunctional Neuropeptides and Hormones in Insects and Other Invertebrates

**DOI:** 10.3390/ijms22041531

**Published:** 2021-02-03

**Authors:** Dick R. Nässel, Shun-Fan Wu

**Affiliations:** 1 Department of Zoology, Stockholm University, S-10691 Stockholm, Sweden; 2 College of Plant Protection, Nanjing Agricultural University, Nanjing 210095, China; wusf@njau.edu.cn

**Keywords:** diuretic hormone, behavior, sleep, feeding, neuromodulation, neurohormone

## Abstract

Leucokinins (LKs) constitute a neuropeptide family first discovered in a cockroach and later identified in numerous insects and several other invertebrates. The LK receptors are only distantly related to other known receptors. Among insects, there are many examples of species where genes encoding LKs and their receptors are absent. Furthermore, genomics has revealed that LK signaling is lacking in several of the invertebrate phyla and in vertebrates. In insects, the number and complexity of LK-expressing neurons vary, from the simple pattern in the *Drosophila* larva where the entire CNS has 20 neurons of 3 main types, to cockroaches with about 250 neurons of many different types. Common to all studied insects is the presence or 1–3 pairs of LK-expressing neurosecretory cells in each abdominal neuromere of the ventral nerve cord, that, at least in some insects, regulate secretion in Malpighian tubules. This review summarizes the diverse functional roles of LK signaling in insects, as well as other arthropods and mollusks. These functions include regulation of ion and water homeostasis, feeding, sleep–metabolism interactions, state-dependent memory formation, as well as modulation of gustatory sensitivity and nociception. Other functions are implied by the neuronal distribution of LK, but remain to be investigated.

## 1. Introduction

Neuropeptide signaling regulates major aspects of development, growth, reproduction, physiology, and behavior of animals. A large number of structurally diverse peptides have been identified that act on different types of receptors as co-transmitters, neuromodulators, and hormones [1,2,3,4,5,6]. In insects, one of the peptides that has attracted substantial attention recently is leucokinin (LK), although it was discovered in a cockroach more than 30 years ago [7]. We thus decided that it is timely to review what we know about LK signaling in insects and other invertebrates.

Like many other well-known insect neuropeptides, LKs were first identified from extract of the head of the Madeira cockroach *Leucophaea maderae* (now *Rhyparobia maderae*) by assaying purified fractions for their activity on hindgut contractions of this animal (see [7,8,9]). Altogether, eight LKs (sequence-related paracopies) were identified in *L. maderae*, which share the C-terminus pentapeptide FXSWGamide [7,10]. Apart from stimulatory action on muscles, another early function assigned to LKs was a role as a diuretic factor that increases secretion in the Malpighian (renal) tubules of various insects [11,12,13,14]. As we shall see in later sections, we now know that LKs have truly pleiotropic functions as neuromodulators and hormones in insect development, physiology, and behavior.

In earlier studies, these peptides were named kinins with a species prefix, such as achetakinins, muscakinins, and lymnokinins, and only later the original name leucokinin was adopted more generally for peptides with the generic C-terminus pentapeptide. Thus, we will use LK here, except when some species-specific aspect is discussed and a species-specific name has been assigned. Early on, it was suggested that the LKs are ancestrally related to the vertebrate tachykinins due to some rather minor amino acid sequence similarities (see, e.g., [7]). Now, with the aid of increased amounts of sequence data and improved bioinformatics tools, we know that LKs and their receptors, LK receptors (LKRs), are not ancestrally related to the tachykinins and their receptors [3,15,16]. In fact, it seems that the LKs and LKRs have no vertebrate orthologs, but they are, with some notable exceptions, present widely among studied arthropods and occur in tardigrades, annelids, and mollusks.

In insects, the genes encoding precursors of LKs can generate varying numbers of isoforms, or paracopies, of LK peptides. Hence, there are from one paracopy in, e.g., *Drosophila* [17,18,19] to 17 in *Periplaneta americana* [20] or even 26 in the western flower thrips, *Frankliniella occidentalis* [21]. The largest number of paracopies was identified in the sea slug *Aplysia*, wherein the LK precursor may give rise to 60 peptides, of which more than 30 are amidated LKs [22]. The first LK receptor (LKR), a G-protein-coupled receptor (GPCR), was identified in the snail *Lymnaea stagnalis* [23]. Later, a cattle tick LKR [24] and a *Drosophila* LKR were characterized [25].

Using antisera to LK-I of *L. maderae*, researchers showed that this peptide is produced by sets of neurons in the cockroach and blowfly brains [26]. Next, it was found that segmentally repeated LK-expressing neurons in abdominal neuromeres of the ventral nerve cord (VNC) are conserved throughout insect species studied [27,28,29,30,31]. These are neurosecretory cells, usually two pairs in each neuromere, with axon terminations associated with peripheral nerves, neurohemal organs, or the body wall of the abdomen. It was suggested that these cells release LK as a circulating hormone that acts on the renal tubules and visceral muscle [27,32]. In contrast to the conserved neuronal expression of LK in neurons of abdominal neuromeres, the brain neurons producing LK are highly variable in numbers and types in different insect species (see [27,28,30,31,33,34]). Thus, one could expect inter-species differences in the complexity of LK signaling in neuronal circuits. Finally, LKs are colocalized with other neuropeptides in some neuronal systems, increasing the plasticity in neuromodulation, also in a species-specific fashion.

This review summarizes different aspects of LK and LKR sequences, distribution, and function in insects. We also highlight LK signaling in other invertebrates, including mollusks, annelids, ticks, and crustaceans and discuss the potential of LKs with reference to peptide analogs in control of pest insects.

## 2. Leucokinins and Their Receptors in Invertebrates

### 2.1. Discovery of Leucokinins and Their Receptors—a Brief History

After their discovery in *L. maderae* in 1986 [7], LKs have been detected in numerous bilaterian invertebrates, but thus far there is no record of related peptides in vertebrates [3,15,35]. Between 1987 and 1999, biochemical identification of LKs from tissue extracts was performed for the locust *Locusta migratoria* [36], cricket *Acheta domesticus* [37], mosquitos (*Aedes aegypti* and *Culex salinarius*) [38,39], housefly *Musca domestica* [40], the moth *Helicoverpa zea* [41], and the vinegar fly *Drosophila melanogaster* [17], as well as the snail *Lymnaea stagnalis* [23].

Early on, immunohistochemistry demonstrated that LKs are produced by both neurons and neurosecretory cells in the brain of *L. maderae* [26]. Next, an analysis of LK distribution in the entire *L. maderae* CNS revealed morphological details of the numerous brain neurons, as well as two pairs of neurosecretory cells in each of the abdominal ganglia [27]. Similar pairs of abdominal LK neurons have been detected in all insects investigated, whereas brain neurons display variations in number and structure [28,29,30,31]. During the mid-1990s immunohistochemistry furthermore suggested presence of LK-like peptides in the spider *Cupiennius salei* [42], the snails *Helix pomatia* and *L. stagnalis* [43,44], and the nematode *Ascaris suum* [45].

The first identification of a gene encoding an LK precursor was derived from analysis of cDNA from the mosquito *A. aegypti* [46], and the first LK receptor was identified and characterized in the snail *L. stagnalis* [23]. Thus, by 1997, it was clear that LKs are encoded by a typical peptide precursor and act on a G-protein-coupled receptor (GPCR) of a new subfamily. Later, an LKR was identified and characterized from a cattle tick [24]. Bioinformatics indicated that this tick LK receptor is related to CG10626 in the *Drosophila* genome [47] and a gene encoding a precursor with a single *Drosophila* LK (CG13480) was subsequently identified [18,19]. The *Drosophila* LKR (CG10626) was finally characterized by means of an in vitro cell expression system [25].

Today, as we shall see in the following sections, LK encoding genes, LK peptides, and their receptors have been identified in multiple organisms from a few different invertebrate phyla. Interestingly, there are quite a few examples of invertebrates that do not seem to possess components of the LK signaling pathway, even among insects. Sequences of LKs from numerous insect species can be gleaned in the DINeR database (http://www.neurostresspep.eu/diner/infosearch, see [48]). This database has 299 sequence entries for LKs from analysis of 86 different insect species, and 50 entries for LK functions in several of these insects. The details of LK occurrence are discussed later.

Early studies demonstrated that LKs stimulate contractions in visceral muscle of the cockroach [9,10,36,49], and that they act in vitro on Malpighian (renal) tubules of several insect species to induce secretion [11,12,13,14,40,50]. Moreover, in *Drosophila,* LK has potent diuretic activity [17,25]. Immunocytochemical localization of LKs in the CNS of insects such as cockroaches and locusts suggests further modulatory roles in interneuronal systems in the antennal lobe, central complex, biological clock circuits, visual system, as well as hormonal roles by means of neurosecretory cells in the brain and abdominal ganglia [26,27,30,34]. In these insects, many of the LK neurons have bilateral arborizations supplying neuropils in both brain hemispheres. About 140 LK immunolabeled neurons were detected in the locust brain and 160 in that of the cockroach [27,34]. In contrast, there are only two pairs of distinct LK neurons in the *Drosophila* brain and each of these neurons is restricted to one hemisphere [28,33]. It is, however, possible that an additional four pairs of neurosecretory cells (designated anterior LK neurons, ALKs) in the brain express low levels of LK (see [33,51]). We discuss the distribution of LK, LKR, and their function in more detail in later sections.

### 2.2. Leucokinins and Their Receptors in Insects and Other Invertebrates—a Comparison between Taxa

**Figure 1:**
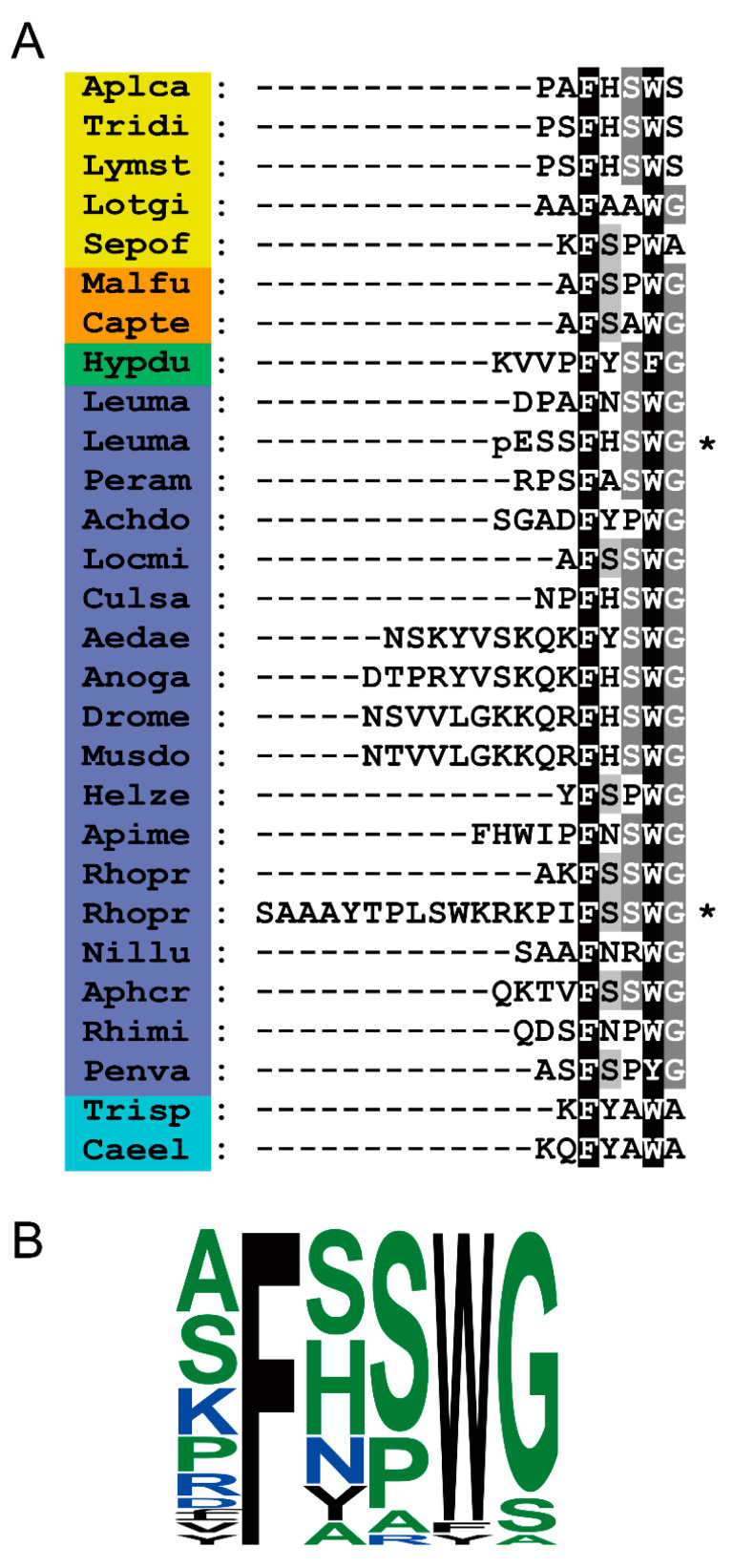
Sequences of leucokinins. (**A**) Sequence alignments of leucokinin and leucokinin-like peptides from select species. Note that C-terminal amidation is not shown. Conserved residues are highlighted in black (identical) or gray (similar). Species belonging to the same phyla have been highlighted with the same color. Species names are as follows, Mollusks: Aplca (*Aplysia californica*), Tridi (*Tritonia diomedea*), Lymst (*Lymnaea stagnalis*), Lotgi (*Lottia gigantea*), Sepof (*Sepia officinalis*). Annelids: Malfu (*Malacoceros fuliginosus*), Capte (*Capitella teleta*). Tardigrade: Hypdu (*Hypsibius dujardini*). Insects: Leuma (*Leucophaera maderae*), Peram (*Periplaneta americana*), Achdo (*Acheta domesticus*), Locmi (*Locusta migratoria*), Culsa (*Culex salinarius*), Aedae (*Aedes aegypti*), Anoga (*Anopheles gambiae*), Drome (*Drosophila melanogaster*), Musdo (*Musca domestica*), Helze (*Helicoverpa zea*), Apime (*Apis mellifera*), Rhopr (*Rhodnius prolixus*), Nillu (*Nilaparvata lugens*), Aphcr (*Aphis craccivora*). Tick (*Ixodida*): Rhimi (*Rhipicephalus microplus*). Shrimp (*Crustacea*): Penva (*Penaeus vannamei*). Nematodes: Trisp (*Trichinella spiralis*), and Caeel (*Caenorhabditis elegans*). Note that the *T. spiralis* and *C. elegans* peptides are unlikely to be LKs as no canonical LK precursor was found (see also text). Sequences indicated by asterisks (*) have special features—in *L. maderae* it is N-terminally pyroglutamate blocked (pE) and in *R. prolixus*, the peptide is N-terminally extended. (**B**) Sequence logo of the LK peptides in different species.

**Figure 2:**
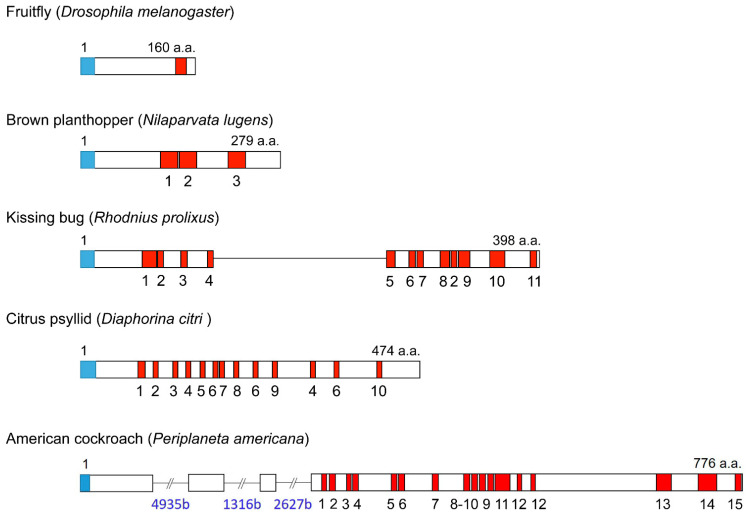
Schemes of organization of representative arthropod leucokinin precursors. Boxes and lines show exons and introns, respectively. Red boxes represent leucokinins, and signal peptides are indicated by blue boxes. The lengths are given in amino acids (a.a.). Primary sequence data of fruit fly, brown planthopper, kissing bug, citrus psyllid, and cockroach were from Terhzaz et al. (1999) [17], Tanaka et al. (2014) [54], Te Brugge et al. (2011) [52], Li et al. (2020) [55], and Zeng et al. 2020 [20], respectively.

Today, there is a wealth of information on LKs and LKRs in invertebrates. In Figure 1, we show amino acid sequences of LKs of some representative invertebrates, including arthropods, a tardigrade, annelids, and mollusks. These LKs are C-terminally amidated and vary in length between 6 and 21 amino acids in common model insects. However, there seem to be some LKs that are even longer (see DINeR database). In insects, the LKs are characterized by an FXSWGamide or FXPWGamide C-terminus, and in other invertebrates FX_1_X_2_WX_3_amide, where X_3_ is S, A, or G. The organization of LK precursors (prepro-LK) display substantial variation, with some (e.g., in *Drosophila*) producing only a single copy of an LK and others multiple copies, for example, 12 in the kissing bug *Rhodnius prolixus,* 26 in the western flower thrips *Frankliniella occidentalis*, and 27 in the bristletail *Meinertellus cundinamarcensis* [17,21,52,53] (Figure 2). Although LKs were first identified in the cockroach *L. maderae*, there is, to our knowledge, no information about the organization of the LK precursor of this species. However, the LK precursor of *Periplaneta americana* has been described [20] (Figure 2 and Figure S1). Interestingly, this precursor can give rise to 17 LK paracopies, but only three of these are identical to LKs of *L. maderae* (Figure S1).

**Figure 3:**
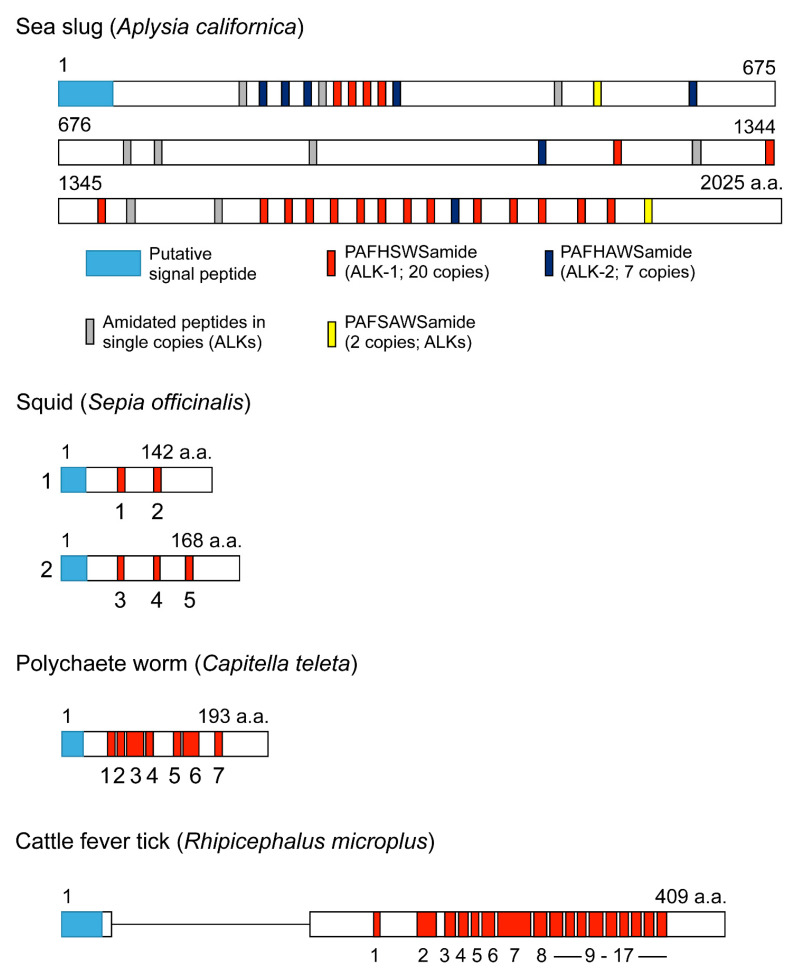
Schemes of representative bilaterian leucokinin precursors. Boxes and lines show exons and introns, respectively. Except for *Aplysia*, red boxes represent leucokinins, and signal peptides are indicated by blue boxes. Primary sequence data of *Aplysia* are from [22], squid from [58], polychaete worm from [15], and cattle fever tick from [59].

Outside arthropods, multiple LK paracopies are also known. In the annelid worm *Urechis unicinctus,* eight paracopies of LKs have been identified [56], and the largest number of LKs was found in the LK precursor of the marine slug *Aplysia californica* with 30 (Figure 3) [22]. In some species, such as *Frankliniella, Rhodnius,* the bed bug *Cimex lectularius*, and *Aplysia*, the prepro-LK can give rise to additional non-LK peptides, resulting in a total of about 60 peptides in *Aplysia* [21,22,52,57]. Of note is that, to our knowledge, none of these non-LK peptides have been studied further in any organism (and very few have been verified by mass spectrometry). There are few cases of a species having more than one LK precursor; one is the squid *Sepia officinalis* with two prepro-LKs [58] (Figure 3).

A striking feature is that in many invertebrate species whose genomes have been sequenced, LK precursors have not been found. Actually, to our knowledge, only arthropods, tardigrades, annelids, and mollusks have thus far been shown to produce LK precursors. Even among insects, not all species have LKs. For instance, in the order Coleoptera (beetles), 34 species have been analyzed, and only in four, *Pogonus chalceus, Gyrinus marinus, Carabus violaceus*, and *Carabus problematicus* (all in suborder Adephaga), LK precursors were found [60,61,62]. Thus, no LK precursor was detected in the “model Coleopteran” *Tribolium castaneum*. LK precursors and receptors are missing also in, for example, some parasitic wasps (e.g., *Nasonia vitripennis*), but not in all [63,64], and were not found in any ant species analyzed to date [65,66,67]. An LK precursor is also missing in the phyllopod crustacean *Daphnia* [68], although they are present in decapod crustaceans (see [69]). It can be noted that an LK-like peptide sequence (Nlp43: KQFYAWAamide) has been identified in nematodes such as *C. elegans* [70] (see Figure 1), but it is not derived from a canonical LK precursor and no LKR could be found [3,15]. Furthermore, LK signaling components are not found in cnidarians (see [71,72]) or flatworms (Platyhelminthes) [73]. In lower bilaterians, such as species of *Xenoturbella* and Nemertodermatid worms (both phylum Xenacoelomorpha), orthologs of LK-type receptors were detected by bioinformatics, but no LK peptides [74]. Finally, in some species, such as honey bees, LK precursors have been identified that could generate three LKs, but the cleaved peptide products could not be detected by mass spectrometry [65]. However, since orthologs of LKRs have been identified in five sequenced bee genomes, it is likely that LK signaling is present in these hymenopterans, but not in ants [65,75]. In support of the importance of LK signaling in honeybees, a recent paper showed that the *lkr* gene influences labor division in foraging for pollen and nectar in the Asian honeybee (*Apis cerana*) [76].

**Figure 4:**
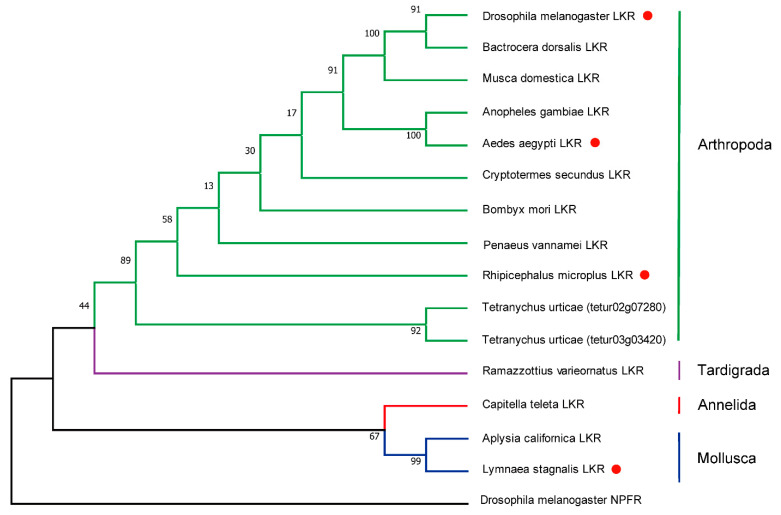
Phylogeny of leucokinin receptors. *Drosophila* neuropeptide F receptor was used as out-group to root the tree. Amino acid sequences of full-length receptors were used for the analysis. Sequences were aligned using the Clustal X. Maximum likelihood trees were constructed by MEGA X software. The numbers at the nodes of the branches represent the percentage bootstrap support (1000 replications) for each branch. Receptors that have been functionally characterized are indicated by a red symbol after the species name. Sequences used to generate the phylogeny are provided in Supplementary Material Text File S1.

As noted above, the LKRs identified seem to have no vertebrate orthologs and are found only in the invertebrate species where LK precursors have been detected (Figure 4), possibly with the exception of Xenacoelomorphs mentioned above. Only a few LKRs have been characterized by ligand activation (Figure 4). Thus, LK signaling is not universally present among invertebrates, in contrast to several other more widespread neuropeptides, such as adipokinetic hormone (AKH)/GnRH, neuropeptide F, and insulin-like peptides (see [1,2,3,15]), and this begs the question as to whether some other neuropeptide system has taken over LK functions. It is also interesting to note the large differences in number of paracopies in the different LK precursors, ranging from 1 to about 30.

## 3. LK Expression Is in Diverse Types of Neurons in the Cockroach *L. maderae* (*R. maderae*)

The distribution of a given neuropeptide in neurons and other cells can provide some initial hints as to whether its functions are diverse or not. Thus, some peptides are present in very small sets of uniform neurons (e.g., SIFamide or eclosion hormone), suggesting few and/or orchestrating functions, and others in large populations of diverse types (such as short neuropeptide F (sNPF) and tachykinins), indicating multiple diverse functions [1,77]. Functional analysis has verified that some neuropeptides are utilized by neurons (and/or other cells) to globally orchestrate development, physiology, or behavior, and others play multiple distributed roles that are more localized and circuit-specific [1,77]. The latter type of peptide action may be in the form of cotransmission, together with other neurotransmitters or neuromodulators [78,79,80]. Therefore, what does the distribution of LKs in different insects tell us about their functions?

**Figure 5:**
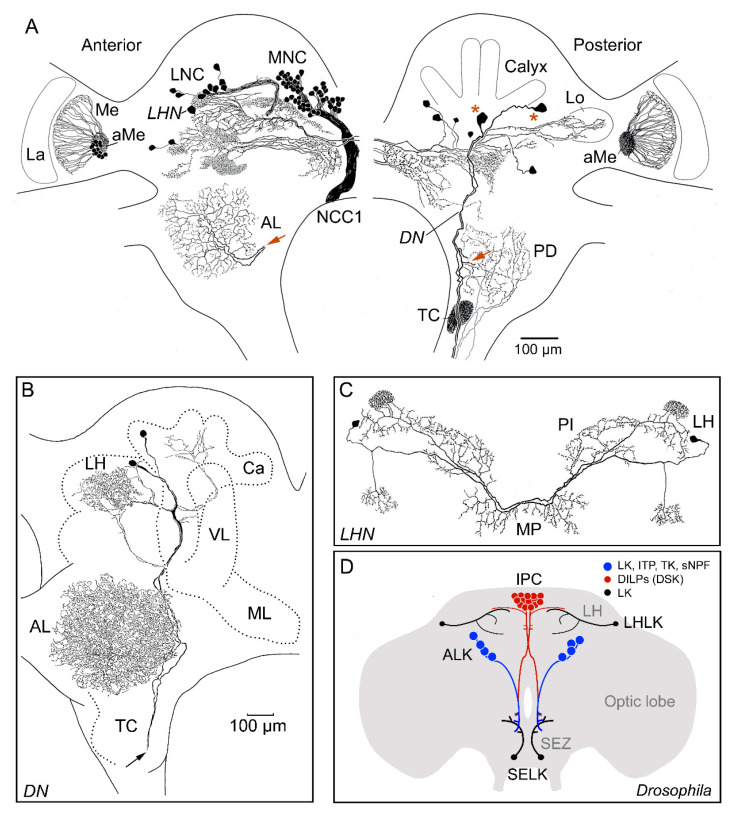
Leucokinin (LK)-expressing neurons in the brain of the cockroach *Leucophaea maderae* (*Rhyparobia maderae*). (**A**) Anterior part of the brain is shown to the left and posterior to the right. Note that the brain contains about 160 LK neurons, all with cell bodies in the protocerebrum. There are LK expressing lateral (LNC) and median neurosecretory cell groups (MNC). The MNCs send axons to the corpora cardiaca via the nerve NCC1 on the contralateral side; the LNCs send axons ipsilaterally via the nerve NCC2 (not shown). A pair or descending neurons (*DN*) in each hemisphere (cell bodies at red asterisks) send axons to the thoracic and abdominal ganglia and have collateral branches arborizing in the antennal lobe (red arrows show where collaterals are). A set of LK neurons have processes in the accessory medulla (aMe) and medulla (Me). A neuron in each hemisphere (*LHN*) has arborizations similar to the LHLKs (lateral horn LK neurons) in *Drosophila*. Other abbreviations: La, lamina; TC, tritocerebral neuropil; PD, posterior deutocerebrum, Lo, lobula. This figure is from [27]. (**B**) Detailed tracing of descending neurons (*DN*) with branches in the antennal lobe (AL), lateral horn (LH), and calyx (Ca) of mushroom body and axons (arrow) running through the tritocerebrum (TC) and circumesophageal connectives to the ventral nerve cord. VL, vertical lobe, ML, median lobe. (**C**) A pair of LK neurons (*LHN*) with branches in lateral horn (LH), pars intercerebralis (PI), and median protocerebrum, resembling LHLKs in *Drosophila* (see panel D). (**D**) Schematic depiction of LK neurons in the adult *Drosophila* brain. LHLKs are located in the lateral horn (LH) and branch extensively in dorsolateral protocerebrum and contact the insulin-producing cells (IPC), known to also produce drosulfakinin (DSK). SELKs (subesophageal LK neurons) are descending neurons with extensive branches in the subesophageal zone (SEZ) and tritocerebrum. ALKs (anterior LK neurons) are lateral neurosecretory cells that only express LK in small and variable amounts in the adult and are known to coexpress ion transport peptide (ITP), tachykinins (TK), and short neuropeptide F (sNPF). Panels (**A**) and (**C**) are from [27] and (**B**) is from [81] (tracing by U. Homberg), all with permission. (**D**) is compiled de novo from data in [33,51,82].

**Figure 6:**
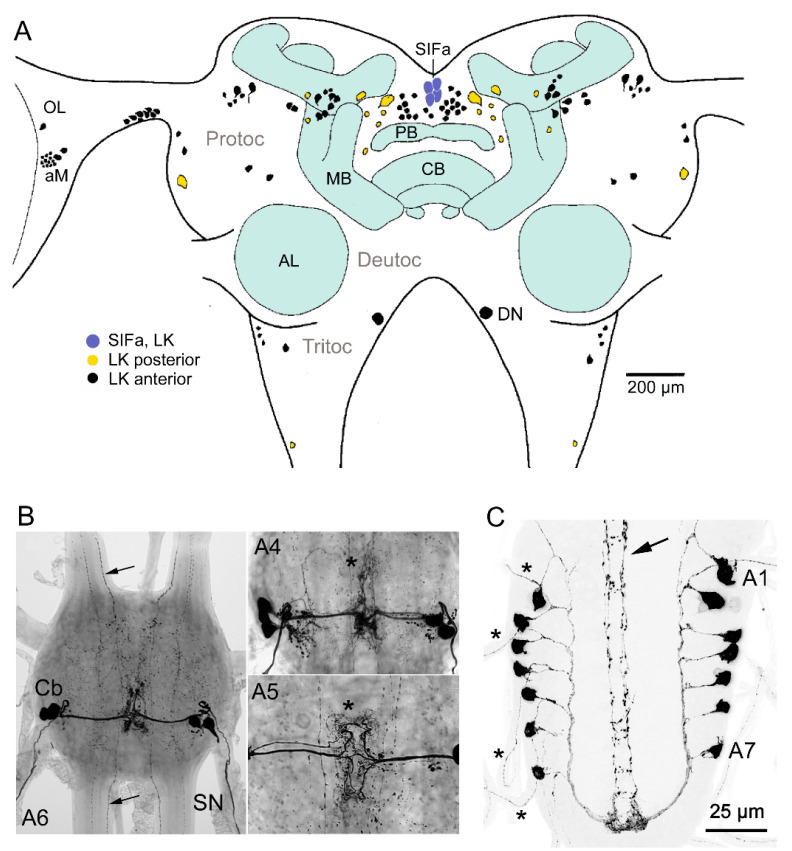
LK neurons in the CNS of the locust. (**A**) LK immunoreactive neurons in the brain of the locust *L. migratoria*. LK cell bodies are predominantly found in protocerebrum (Protoc), including the optic lobes (OL) and accessory medulla (aMe; pacemaker region of clock), but some are in tritocerebrum (Tritoc). Neuronal process from LK neurons (not shown) are in the central body, optic lobe, and antennal lobe (AL), and less delineated neuropils are shown in all three brain neuromeres. A group of four neurons (SIFamide producing (SIFa)) in the pars intercerebralis coexpress SIFamide and LK. These SIFa neurons are known to send processes throughout the brain and ventral nerve cord [83,84] similar to the four SIFa neurons in *Drosophila* [85]. (**B**) LK immunoreactive neurons (neurosecretory cells) in abdominal ganglia of the locust *Locusta migratoria*. In more anterior abdominal ganglia (A1–A4), there are three pairs of LK neurons, and in the posterior ones (such as A6 shown here), there are only two pairs. The arrows depict axons from the descending neurons (a total of four axons). Other abbreviations: Cb, cell body; SN, segmental nerve. (**C**) In larval *Drosophila*, there is one pair of LK immunoreactive ABLKs (abdominal ganglion LK neurons) in each of the abdominal neuromeres A1–A7. These send axons to the body-wall muscle via segmental nerves (asterisks). Arrow indicates axons of the two pairs of descending neurons, SELK. Panel A is altered from [34] with SIF neurons added [83], B is from [86], and C is altered from [87]. All figures used with permission from publishers.

The distribution of LKs was first analyzed in *L. maderae*, using antisera to LK-I that recognized the eight isoforms known at the time [26,27,32]. In this cockroach, the number and diversity in cell types expressing LKs is large, suggesting a wide range of functions for this set of peptides (Figure 5A–C). Thus, we used these old LK immunohistochemical data to illustrate a peptidergic system quite different from that in *Drosophila* (see Figure 5D) and some other insects, such as locusts (Figure 6A).

There are about 160 LK neurons with cell bodies in the protocerebrum of the brain (Figure 5A), some in bilateral clusters and others occurring in bilateral pairs distributed in different regions [27]. No cell bodies were detected in deuto- and tritocerebrum, and only a small set of weakly immunoreactive neurons were detected in the fused subesophageal ganglion. In each of the two lateral neurosecretory cell (LNC) groups there were six LK cells, and in the median neurosecretory cell group (MNC), about 100 were found (Figure 5A). Both the LNCs and MNCs send LK-immunolabeled axons to the neurohemal area of the corpora cardiaca, suggesting that the LKs can be released as hormones into the circulation. Radioimmunoassay analysis of HPLC-separated corpora cardiaca extracts suggested that all eight LKs known at the time are present in this tissue [32]. Furthermore, it was indeed shown by radioimmunoassay (RIA) that release of LKs can be triggered in vitro from the corpora cardiaca of both cockroach [88] and cricket [89]. Furthermore, in the bug *Rhodnius*, RIA of hemolymph demonstrated both LK and diuretic hormone (DH44) release after feeding, suggesting a postprandial hormonal role of LK [90].

In contrast to *Drosophila* where one pair of LK interneurons is seen in the brain and one pair in the subesophageal zone (SEZ) (Figure 5D), the cockroach brain has a complex set of interneurons (Figure 5A–C). Different LK neurons, originating in the protocerebrum, send processes to the central body; optic lobe (medulla and lobula); antennal lobes; and to various neuropil regions in the proto-, deuto-, and tritocerebrum. Two pairs of large LK-immunoreactive descending neurons (DNs) send axons throughout the ventral nerve cord, finally ending in the terminal abdominal ganglion. These pairs of DNs have collateral arborizations ipsilaterally in most of the glomeruli of the antennal lobe and posterior deutocerebrum (Figure 5A,B). A small set of branches from the DNs innervates the calyces of the mushroom bodies [81] (Figure 5B). The group of LK neurons associated with the medulla [27] has been described in more detail as part of the accessory medulla complex that is a pacemaker region of the circadian clock [91,92,93]. Some of these LK neurons colocalize pigment-dispersing factor (PDF), which is one of the major neuromodulators of the clock in *L. maderae* and *Drosophila* [91,93,94].

Similar to *Drosophila* and other studied insects, each abdominal ganglion has sets of neurosecretory cells (ABLKs; abdominal ganglion LK neurons) expressing LK. However, instead of one pair of ABLKs in each ganglion/neuromere, as seen in *Drosophila* and some other dipteran flies [28], *L. maderae* has two pairs [27]. Two pairs of ABLKs are also seen in, e.g., crickets, crane flies, moths, and mosquitos, whereas there are three pairs in the first four abdominal ganglia of locusts and two in the following ganglia (Figure 6B) and up to 10 pairs per ganglion in dragonflies [29,30,95,96]. In cockroaches and locusts, these ABLKs send varicose axons to the lateral heart nerves and transverse nerves, where neurohemal areas (perivisceral organs) are formed; moreover, spiracles receive LK axon terminations [27,30,97]. Although LK was originally isolated by means of its activity on hindgut contractions, no LK innervation of this tissue was detected [27], suggesting that this myotropic action is mediated by hormonal LK. Another difference to *Drosophila* is that the cockroach thoracic ganglia each have at least two pairs of LK-expressing interneurons that arborize widely in the lateral portions of the ganglia [27,30].

As in *Drosophila,* there are no LK expressing enteroendocrine cells (EECs) in the *L. maderae* intestine. However, there are bi- or multipolar LK neurons in the posterior midgut with ascending axons running via the esophageal nerve to end with arborizations in the frontal ganglion and tritocerebrum [27]. These might be proprioceptive cells that signal gut distension to the frontal ganglion and other feeding circuits. Additionally, LK-immunoreactive axons from the retrocerebral complex (in particular the frontal and hypocerebral ganglia) were found to innervate the pharynx and esophagus [27]. Mapping of LK neurons in the brain of the cockroach *Nauphoeta cinerea* revealed a similar set of neuron types [30].

Thus, taken together, the cockroach LK neurons are more diverse than those in *Drosophila* (Figure 5A–D) and seem to underlie distributed functions in different brain/ganglion regions. Such functions may include neuromodulation in the olfactory system, visual system, central complex, mushroom bodies, circadian clock, tritocerebral neuropil, circuits of the thoracic ganglia, and the frontal ganglion (regulation of feeding) [27]. The two pairs of protocerebral descending LK neurons (Figure 5A,B), which span the entire ventral nerve cord, may provide a pathway for linking protocerebral and olfactory systems to regulate ganglionic activity. In addition, there are three types of neurosecretory cells producing LKs, namely, LNCs, MNCs, and the ABLKs, which probably release LKs into the circulation to target peripheral organs such as Malpighian tubules, heart, and visceral muscle. Furthermore, peripheral cells were found in the intestine of *L. maderae* that may be proprioceptors.

Unfortunately, there are no data on any functions of LKs in cockroaches, except the stimulatory activity on the hindgut muscle in vitro [7,9]. Thus, we can only speculate that LK signaling in the cockroach is functionally more diverse than in *Drosophila* with its four neurons in the brain/SEZ and 22 ABLKs. The four brain/SEZ neurons of *Drosophila* (Figure 5D) do not seem to have any obvious analogs in the cockroach brain, but there are three bilateral pairs of *L. maderae* LK neurons that could play roles similar to the pair of LHLKs (one is labeled LHn in Figure 5A,C). The SELKs are descending neurons in *Drosophila* with cell bodies and processes in the SEZ [33,51], whereas the cockroach descending neurons originate in the protocerebrum and innervate the antennal lobes on their descent (Figure 5A,B). The LK-expressing LNCs of *L. maderae* may be analogous to the ALKs of *Drosophila* (Figure 5D). These *Drosophila* ALK neurons can be seen in several *Lk*-Gal4 lines, but only in early larvae do they consistently label with antisera to LK [33,51]. These *Drosophila* neurons were first described as LNCs expressing ion transport peptide (ITP), a peptide that may act in regulation of thirst and hunger and probably also plays a role in ion transport in the intestine [98,99]. The *Drosophila* ALKs were also shown to express tachykinins (TKs) and short neuropeptide F (sNPF), and these peptides were found to regulate metabolic and desiccation stress responses [82]. It is not known whether the *L. maderae* LNCs express further neuropeptides, but possibly their functional roles are similar to those of *Drosophila*. On the basis of the anatomy and distribution of the cockroach LK neurons, one could speculate that some of the other LK functions determined in *Drosophila* also apply to *L. maderae*—roles in the circadian clock output and sleep, in feeding, and in regulation of water and ion homeostasis (see [17,51,100,101,102,103]).

## 4. Distribution of LK in Other Invertebrates: What Can Comparative Studies Teach Us?

In the previous section, we described the LK neurons of the cockroach *L. maderae* with some comparative comments on *Drosophila,* two insects that highlight two extremes in terms of number and diversity of LK neurons. Here, we briefly summarize findings of interest in other invertebrates and discuss coexpression of LK and other peptides.

### 4.1. LK in Neurons of the Brain of Other Insects

LK distribution has also been described in the brains of several other insects, including the blood-sucking bug *Rhodnius prolixus*, the locusts *Locusta migratoria* and *Schistocerca gregaria,* the cricket *Acheta domesticus*, and the mosquito *Aedes aegypti* [30,31,34], which is summarized in Table 1. As an example, we show LK neurons in *L. migratoria* (Figure 6A), where some interesting features differ from *Drosophila* and *Leucophaea*.

The distribution of various neuropeptides has been extensively investigated in the locust brain, some in exquisite detail (see [104,105]), whereas the LK distribution has received more superficial attention. In the brain of *L. migratoria*, about 140 LK immunoreactive neurons were detected [34] (Figure 6A). Their cell bodies are primarily located in the protocerebrum, but about 5–6 pairs were detected in the tritocerebrum. No clear-cut neurosecretory cells were seen in the brain, but LK-expressing interneurons are associated with the optic lobe and the accessory medulla (pacemaker center of the clock), the central body, and antennal lobe [34,105]. As in the *L. maderae* brain, two pairs of descending LK neurons innervate the antennal lobes on their way to the ventral ganglia in *S. gregaria* [34,106]. There is an additional pair of larger tritocerebral descending neurons in *L. migratoria* [34] (Figure 6A). Distinct LK immunolabeled processes can be seen in protocerebral neuropils such as the upper and lower divisions of the central body, the median and lateral accessory lobes of the central complex, and the protocerebral bridge, but not in the mushroom bodies. In the optic lobes, specifically the most basal portion of the lamina, different layers of the medulla (including the accessory medulla) and lobula contain LK fibers. A supply of immunoreactive fibers can also be seen in the glomeruli of the antennal lobe and many of the non-glomerular neuropils of proto-, deuto-, and tritocerebrum contain diffusely arborizing LK fibers.

An interesting finding is that in *S. gregaria* a set of four SIFamide-expressing neurons in the pars intercerebralis of the brain colocalize LK [83] (see Figure 6A). As in *Drosophila*, the processes from these SIFamide neurons innervate most neuropil regions of the brain and ventral nerve cord [83,85]. The LK expression in these neurons is weak in adult locusts, but nevertheless suggests that LK may play a role in the signaling of these SIFamide neurons. The homolog SIFamide neurons in *Drosophila* are known in to orchestrate feeding, sleep, and mating in a nutritional state-dependent fashion [85,107,108]. Another interesting aspect of these SIFamide neurons in the locust is that they are identical to the LK-expressing primary commissure pioneer neurons (PCPs) that lay down an early axonal tract (commissure) in the brain of the locust embryo [83,84]. Since the LK immunolabeling was found stronger in the SIFamide neurons in younger stages than in the adult [84], it is suggestive that LK plays a role of during neuronal development and axonal pathfinding in the brain.

In the brains of the cricket *Acheta domesticus* and the mosquito *Aedes aegypti,* the distribution of LK neurons is similar to that in *L. maderae*, with both LNCs and MNCs and their axon terminations in the corpora cardiaca expressing the peptide, but other interneurons were not described in enough detail for comparisons to be made [30]. The same authors found that there are no LK-immunoreactive neurons in the brain of the honeybee *Apis mellifera*, but only neurosecretory cells in the abdominal ganglia [30].

Finally, in the brain of the blood-sucking bug *Rhodnius prolixus,* about 180 pairs of LK-immunoreactive neurons were detected, 30 pairs of which were more strongly labeled [31]. These were later confirmed by in situ hybridization [109]. Processes of LK interneurons were seen widely in brain neuropils. In starved specimens, a set of MNCs and their processes in the corpora cardiaca could be detected with LK antiserum [31], suggesting LK expression is dependent on nutritional state and that this peptide plays a role as a systemic hormone. Injection of a biostable analog of an LK displayed decreased intake of blood in a feeding assay [110]. Furthermore, RIA of hemolymph demonstrated that LK is released after feeding [90]. In *R. prolixus*, LK does not display diuretic activity in the Malpighian tubules or anterior midgut (in contrast to, e.g., DH44), but it decreases the resistance and transepithelial voltage of the epithelium and also increases the frequency of contractions in the anterior midgut [31,111]. LK also induces contraction in the *R. prolixus* hindgut [109,110]. *R. prolixus* is the only insect that has thus far displayed LK-producing enteroendocrine cells in the midgut [31].

### 4.2. LK in Neurons of the Nervous System of Other Invertebrates

The only phylum outside arthropods where bona fide LK distribution has been described is in mollusks. LK-expressing neurons in mollusks have been mapped for *Lymnaea stagnalis, Helix pomatia*, and *Aplysia californica* [22,44,112].

In the snail *Helix*, about 700 LK immunoreactive neurons were found in the CNS [112]. Buccal, cerebral, and pedal ganglia, as well as the viscero–parietal–pleural ganglion complex, all express LK in numerous neurons. One giant LK neuron was found in the pedal ganglion. Two major groups of LK neurons in the cerebral ganglia send axons into commissures to other ganglia and into several peripheral nerves [112]. Several peripheral tissues such as buccal mass, oviduct and intestinal muscle, and “skeletal” muscle (of foot, lip, and tentacle) are supplied by varicose LK axons. In addition, bipolar LK neurons were found in the intestine and were shown to send axons into the extensive meshwork of LK fibers seen there. Some groups of LK neurons in the cerebral ganglion coexpress tachykinin immunoreactivity [112]. It is not clear whether any of the LK neurons serve as bona fide neurosecretory cells, but it cannot be excluded that the abundant superficial LK axons in peripheral tissues might release LK into the circulation.

In *Aplysia*, the majority of the LK neurons were found in the buccal ganglion, which is known to house feeding motor neurons and pattern-generating interneurons [22]. LK neurons were also seen in the cerebral ganglion, where higher-order feeding interneurons are located. These authors found that the buccal motor neuron B48 expresses LK and that application of this peptide ex vivo modulated a parameter of the consummatory feeding behavior [22]. One target of LK action is a central pattern generator element that modulates the duration of the protraction phase of feeding responses. Thus, this *Aplysia* study provides a mechanistic description of LK modulation of food ingestion, something that is lacking thus far for *Drosophila* and other insects. However, roles of LK in food consumption and post-feeding physiology have been demonstrated in *Drosophila* [51,103,113] and are suggestive in *Rhodnius* [31,111].

### 4.3. Neurosecretory Cells and Hormonal Roles of LK in Invertebrates

One striking conserved feature is that all studied insects have segmental abdominal neurosecretory cells (ABLKs), varying in number between one pair per neuromere in *Drosophila* (Figure 6C) and blowflies, to up to 10 pairs in dragonflies [28,29,30,31,96]. Commonly insects have two to three pairs per neuromere/ganglion (see [29,30,97]) (Figure 6B). These neurosecretory cells have axon terminations associated with peripheral nerves (including lateral heart nerves), perisympathetic organs, and the body wall muscle of the abdomen. Since these abdominal cells are the only LK-expressing neurosecretory cells in several species studied, it is suggestive that these cells release LK as a circulating hormone. Thus, an important function of LKs is as hormones that act systemically, as diuretic factors, and that they are also likely to regulate gut contractions in some species (see [9,11,36,49,50,75,87,114,115]). As mentioned, LK release has been demonstrated in *L. maderae, A. domesticus*, and *R. prolixus* [32,89,90]. In several insect species, including *Drosophila*, *Musca domestica, Manduca sexta,* and *Rhodnius prolixus*, the abdominal LK cells coexpress the neuropeptide DH44 [31,87,116,117]. In *Rhodnius,* the DH44 stimulates secretion in the Malpighian tubules, whereas LK has no direct action on tubules, but may act elsewhere (e.g., anterior midgut and hindgut) to assist in rapid diuresis [31,110,111]. Both LK and DH44 are released after feeding in *Rhodnius* [90]. In *Drosophila*, on the other hand, both DH44 and LK stimulate secretion in the tubules, but by acting on different cell types and with different signal pathways downstream the receptors [17,75,87,118,119].

As mentioned above, some insect species possess additional LK-expressing neurosecretory cells systems in the brain. It is not known whether the cells of the brain and the abdominal ganglia (when both exist) play different functional roles, but it is at least likely that LK release in these cell groups are under control by different central neuronal circuits. It is also possible that in the LNCs other neuropeptides are colocalized with LK, as is the case in the *Drosophila* ALKs with additional TK, ITP, and sNPF [51,82]. For instance, in *M. sexta* and *L. migratoria*, sets of LNCs are known to produce ITP [120,121]. Interestingly, the only insect known that has LK-expressing endocrine cells in the midgut is *R. prolixus* [31]. Thus, LK is a rare peptide in intestinal signaling, in contrast to many other neuropeptides (see [122,123,124]).

In crustaceans, LKs have not yet been detected in the canonical neurosecretory system, the X-organ/sinus gland of the eyestalks, or in the stomatogastric system [125]. However, in pericardial organs of the crab *Cancer borealis*, varicose LK immunoreactive axons were detected (probably derived from cell bodies in thoracic ganglia), suggesting that hormonal release of LK is possible [126]. Peptides from the pericardial organs are known to act as circulating hormones on circuits of the crab stomatogastric ganglion, and indeed shrimp LK applied to the ganglion has a distinct modulatory action on the pyloric rhythm of the network [126].

Not all arthropods seem to use LKs as hormones. In the spider *Cupiennius salei*, no LK-immunolabeled neurosecretory cells were detected, and actually the LK interneurons are not segmentally arranged, but the nine pairs of cell bodies are clustered anteriorly in the supraesophageal ganglion [42]. However, in another arachnoid, the tick *Rhipicephalus appendiculatus*, four pairs of neurosecretory cells located anteriorly in the prothocerebral lobe produce LK [127]. These cells have arborizing axon terminations in neurohemal areas in the neural sheath surrounding the CNS, and colocalize the neuropeptide myosuppressin.

In mollusks, no bona fide neurosecretory cells producing LK have been described, but in the snail *H. pomatia*, sets of LK neurons clustered in cerebral ganglia have axons running out in several nerve roots to innervate peripheral tissues [112]. These peripheral varicose axons might release LK into the circulation, but further studies are required to verify this.

Although LK has been demonstrated in annelids, such as *Urechis unicinctus* and *Capitella teleta* [56,128], there are, as far as we know, no reports on the cellular localization of the peptide. In the parasitic nematode *Ascaris suum*, LK immunoreactivity was detected in neurons [45], but no LK precursor gene has been identified in nematodes, and thus it is not clear what endogeneous peptide the antiserum recognized.

### 4.4. Specific Roles of LK Signaling in Arthropods

Here, we present a brief summary of the diverse functions of LK signaling in arthropods. Most of the recent work has been performed in *Drosophila*, but we will describe that only very briefly in Section 4.4.5, since a more detailed review on *Drosophila* will appear elsewhere. In Table 2, we list known functions of LK signaling in different insects and some other invertebrates.

#### 4.4.1. Myostimulatory Action

LKs act in vitro to increase frequency and amplitude of contractions in the hindgut of *L. maderae* [7,9] and the housefly *Musca domestica* [40], and in the anterior midgut and hindgut of the bug *R. prolixus* [109,110,111], but have no effect on neither hindgut nor oviduct contractions in the locust *L. migratoria* [36,49].

#### 4.4.2. Diuretic Action

A more widespread action is the stimulatory action of LKs on Malpighian tubules shown in [11,12,14,17,41,46,50,75,97,116]. In the studied insects, LKs activate the LKR, leading to an increase in intracellular calcium, which activates a chloride shunt conductance and water transport across the tubule epithelium [14,118,145,146]. In dipteran insects, such as *Drosophila, Anopheles gambiae*, and *Aedes aegypti,* this action is mediated by stellate cells of the tubules, which express the LKR [25,75,118,147]. LK signaling appears secondarily lost in most species of beetles (Coleoptera), and mining of the genome of *Triboleum castaneum* shows that other signaling systems known to be associated with diuretic functions in insects are greatly expanded [75].

#### 4.4.3. Modulation of Sugar Gustation in the Mosquito *Aedes aegypti* and Asian Honeybee *Apis cerana*

In females of the mosquito *Aedes aegypti*, application of a protease-resistant LK to the mouthparts and proleg tarsi resulted in inhibition of sucrose feeding and induction of an escape behavior, wherein the insect walked or flew away from the food [138]. It was shown that the LKR is expressed in chemosensory cells in proleg tarsi and labellar sensillae, and LK analog applied to mouthparts blocked the electrophysiological response to sugar in chemosensory sensillae. Furthermore, LKR-RNAi (RNA interference) by injection of double-stranded RNA eliminated the inhibitory effect of LK on sugar feeding [138]. This effect of a stable LK analog suggests a promising lead for a feeding deterrent in control of mosquitos as disease vectors [138]. Moreover, in the Asian honeybee *A. cerana*, sucrose-sensing is modulated by LK signaling [76]. Knockdown of the LKR by RNAi decreased the sensitivity to sucrose in a proboscis extension response assay. Furthermore, the *Lkr* gene influences division of labor in foraging in these bees, and nectar foragers display lower Lkr expression than those foraging for pollen [76].

#### 4.4.4. Feeding and Fecundity in the Cattle Fever Tick

In the cattle fever tick *Rhipicephalus microplus*, silencing of the LKR by double-stranded RNA injection induced decreased egg production and hatching of eggs laid, and also delayed oviposition [144]. This effect appears to be indirect since the authors did not report expression of the LKR in ovaries but did report expression in the outer muscle layer of the midgut [144]. It was suggested that LK action on the gut affects gut motility and potentially uptake and processing of nutrients, and this in turn affects nutrient availability and fecundity [144]. An inhibitory effect of LKs on release of the digestive enzymes protease and amylase from the midgut was in fact shown in the moth *Opisina arenosella* [142], and myostimulatory effects of LKs are known in several insects [8,9,111]. It is possible that the LK action in the tick also includes the CNS, which could affect control of feeding and/or hormone release that reduces reproductive output.

#### 4.4.5. Feeding in *Rhodnius prolixus* and *A. aegypti*

In *R. prolixus* and females of the mosquito *Aedes aegypti*, protease-resistant LK analogs reduce food intake when injected in the former and applied to the mouthparts and proleg tarsi of the latter [110,138]. Thus, LKs can have anti-feedant activity.

#### 4.4.6. Functional Roles of LK in *Drosophila*

**Figure 7:**
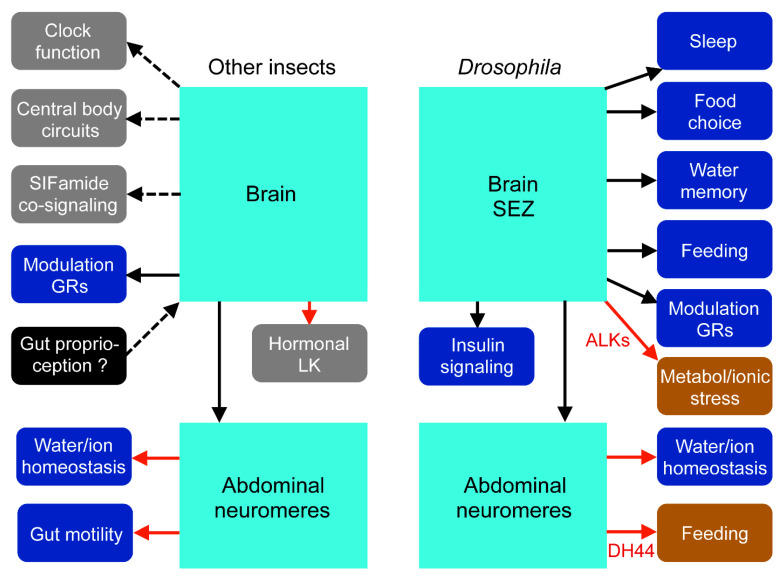
Summary of LK functions in *Drosophila* compared to other insects. In insects other than *Drosophila*, few functions have been explicitly determined (blue boxes), and most are suggested from LK expression (grey or black boxes). Red arrows indicate hormonal signaling, black arrows indicate established functions, and dashed arrows indicate suggested functions. In the mosquito *A. aegypti*, LK regulates sugar taste in gustatory receptor neurons (GRs) [138]; in the cockroach *L. maderae* and some other insects, intestinal contractions are regulated by LK; and in many insects, LK acts as a diuretic factor [75,133]. LK-expressing sensory cells in the intestine of *L. maderae* send axons to the frontal ganglion and brain, suggesting proprioceptive inputs [27]. In *L. maderae*, LK is present in pacemaker neurons of the clock circuit [27,93], and in the locust *L. migratoria*, LK is expressed in the four widely arborizing SIFamide-producing neurons and in circuits of the central body [34,83]. In several insects, including *L. maderae*, there are LK-expressing lateral and median neurosecretory cells indicating hormonal LK signaling from the brain [27,30,31]. In *Drosophila*, genetic interventions have revealed actions of specific neurons in the brain, subesophageal zone (SEZ), and abdominal neuromeres in several functional roles (blue boxes). These are metabolism–sleep interactions [100,101,102,103], food choice [131], water- and sugar-enforced memory [130], food intake, modulation of GRs [139,141], and water and ionic homeostasis [51,87,103]. One set of LK neurons (LHLKs) also regulates insulin-producing cells [51,103]. LK neurons expressing additional peptides contribute to other functions with non-LK peptides (red boxes). These are the ALK neurons that signal with LK, ITP, sNPF, and TKs and regulate metabolic and ionic stress responses (as well as feeding and drinking) [82,98], and ABLKs that also express DH44 and this peptide affect feeding and water balance [87].

In recent years, *Drosophila* studies have employed genetic interventions and have revealed actions of specific LK neurons in the brain, SEZ, and abdominal neuromeres (Table 2, Figure 7). The two LHLK neurons (Figure 5D) were shown to modulate metabolism–sleep interactions and serve as clock output [100,101,102,103], modulating state-dependent water and sugar-enforced memory [130], and probably food choice [131]. This pair of LHLK neurons also regulates insulin-producing cells, which may contribute to sleep–metabolism effects [51,103]. The abdominal ABLKs (see Figure 6C) regulate water and ionic homeostasis along with associated stress [51,87] and mechanosensory-induced defensive post-mating response in females [132]. Moreover, in *Drosophila,* LK modulates gustatory neurons, but it is not clear which neurons are responsible [139,141], although the SELKs are in a favorable position. The ABLKs co-express DH44 and specific knockdown of this peptide in ABLKs affect water and ionic homeostasis, as well as feeding [87]. The ALK neurons (Figure 5D) are likely to signal with LK, ITP, sNPF, and TKs [51,82]. The function of LK in these cells is not yet known, but sNPF and TKs regulate metabolic and ionic stress responses [82], and ITP modulates water and ionic homeostasis, as well as feeding and drinking [98]. As seen in Figure 7, some of the functions of LKs appear conserved between *Drosophila* and other insects: clock-sleep functions, modulation of gustatory neurons, regulation of water and ion homeostasis, and possibly feeding.

## 5. Targeting the LK Signaling System with Peptide Analogs to Aim at Pest Control

Neuropeptides regulate many vital processes in the daily life of insects such as development, growth, feeding, reproduction, metabolism, and water and ion homeostasis. These roles, taken together with the high specificity and activity at very low doses, render neuropeptides and their cognate receptors potential leads for the development of eco-friendly insecticidal agents [148,149,150,151,152,153,154,155]. Of the different peptides known, LKs have received considerable attention since the LK/LKRs signaling system seems to have no vertebrate orthologs and it plays a key role in regulation of many vital physiological and behavioral processes in insects, as shown in Section 4.4. In insects, LKs are multifunctional neuropeptides that share a common C-terminal pentapeptide sequence FX_1_X_2_WGamide, where X_1_ can be H, N, S, A, or Y and X_2_ can be S, P, A, or R (see Figure 1B); this pentapeptide is also the active core of LKs, facilitating peptide design [40,152,156]. As noted in a previous section, LKs have been identified a wide range of insects (see the DINeR database: http://www.neurostresspep.eu/diner/infosearch), with the exception of most beetles (Coleoptera), all ants, and some wasps (Hymenoptera) [60,62,63,64,65,67]. Since LKs are rapidly degraded by peptidases, analogs of insect LKs have been synthesized with a modified chemical structure to increase stability [152,156]. Replacement of the X_2_ residue of the C-terminal pentapeptide core sequence (FX_1_X_2_WGamide) with an alpha-aminoisobutyric acid (Aib) resulted in resistance to hydrolysis by angiotensin-converting enzyme (ACE) and neprilysin (NEP) [157,158]. A rationale for this is that the X_2_ position is the primary site of susceptibility to peptidase cleavage. Incorporation of a second Aib residue adjacent to the secondary peptidase hydrolysis site (N-terminal to the F residue) further enhances biostability [157]. These short LK analogs have activities that are similar to or exceed those of native insect kinins when tested on recombinant LKRs from the southern cattle tick *Rhipicephalus microplus* and the dengue vector, *Aedes aegypti* [59,159,160,161]. Both in tissue bioassays and in recombinant LKR experiments in vitro, it was shown that that the F residue (in position one), W (in position four), and the amidated C-terminus of the LK pentapeptide core are crucial for LK activity [159,160,162]. Some modified biostable insect LK analogs have potential to be used in the integrated pest management because they reduce gain in body weight in corn earworm *Helicoverpa zea* larvae [157,163] and increase aphid mortality [164,165,166]. A biostable LK mimetic, (analog 1728; K-Aib-1), was shown to inhibit sugar taste receptors and act as a feeding deterrent in *Aedes aegypti* mosquitoes [138]. Moreover, in the bug *R. prolixus*, a stable LK analog displayed antifeeding activity after injection [110], and induced increased activity on hindgut contractions [109]. In female ticks, knocking down the expression of the LKR leads to a significant reduction of their reproductive fitness [144]. Hence, the tick LKR might be a promising target for developing more potent analogs. A recent study screened 14 predicted *R. microplus* LKs (Rhimi-K) and 11 LK analogs containing Aib and found that all of them were full agonists and displayed potent effects on the LKR of *R. microplus* [59]. These tick LKs and LK mimetics provide putative tools for tick physiology and management. However, the practical exploitation of the insect and tick LKs and LKRs for pest control is still in its early stages. More work is needed to solve the bio-stability, cost of production, and bio-safety of neuropeptide analogs, as well as to find efficient modes of peptide administration to target pest insects.

## 6. Conclusions

In this review, we have shown that expression of LKs is variable among invertebrates. Not only is it absent in many taxa, including some insect groups, but also its cellular expression varies between different insect species. Thus, there are 20 LK neurons of 3 major types in the CNS of the *Drosophila* larva (plus the enigmatic ALKs) and about 250 of multiple types in that of adult *L. maderae* [27,33]. A conserved feature is, however, the segmentally arranged neurosecretory ABLKs found in all insects studied (see [27,30,31,95]). This suggests that a hormonal role of LKs is a conserved feature among insects, and that a common action is to induce secretion in the Malpighian tubules [50,75] and potentially action on contractility and epithelial transport in the gut [9,111]. Most other functional roles of LK have been studied only in *Drosophila*, and thus it is not clear at this point to what extent further functions are conserved. However, as seen in Table 2, regulation of taste receptors and feeding, signaling in clock and sleep circuits, as well as gut function may be outputs of LKs in several invertebrate species.

Interestingly, even amongst insects, genes encoding LK and LKR are lacking in many species. Is the lack of LK signaling compensated somehow? A clue can be obtained from looking at diuretic functions in beetles (Coleoptera) where most species have no LK signaling components. In the beetle *Tenebrio molitor,* genes encoding other diuretic hormones and their receptors (and associated downstream molecules) are upregulated, suggesting that peptide hormones are interchangeable to some extent [75]. This is also emphasized by the fact that LKs are strong diuretic factors in some insect species such as *Drosophila* and mosquitos, but have no direct action on diuresis in, e.g., *Rhodnius* [17,31,111]. Regulation of water and ion homeostasis is complex, with several peptide hormones involved [1,50,114,133]. In locusts and *Drosophila*, colocalized LK and DH44 activate different signaling systems downstream of their receptors, but act synergistically to induce secretion in tubules [87,114]. The interactions between LKs and other diuretic and antidiuretic hormones are not yet known, but it is likely that hormonal regulation of water and ion balance differs between different taxa both in terms of hormones involved and cellular mechanisms.

Moreover, in the CNS, functions of LKs may be carried out by other neuropeptides when LKs have been lost (or never evolved), but what could be the significance of the larger number and diversity of LK neurons in the cockroach brain compared to that of *Drosophila*? Many neuropeptides act as local neuromodulators, often as cotransmitters of small-molecule neurotransmitters [78,79,80,167]. Thus, it is likely that LK produced in smaller interneurons of *L. maderae* serve local neuromodulatory/cotransmitter roles, similar to, for instance, TKs and sNPF in *Drosophila* [16,78,168,169]. In *Drosophila*, on the other hand, the four LK interneurons in the brain/SEZ have relatively wide arborizations and seem to play roles in orchestration of physiology and behavior. Clearly, we need more experimental data from other insects to be able to understand core functions of LK signaling and to further appreciate how some functions may have diversified during evolution. Finally, as described in the previous section, LKRs have been chosen as candidate targets for development of stable peptide mimetics for use in insect and tick pest control. Perhaps also development of small molecule ligands of LKRs would be useful in this quest to interfere with the vital LK signaling.

## Figures and Tables

**Table 1:** Distribution of LK neurons in the brain of representative insects ^1.^

Species	CB	AL	OL	LH	TC, SEZ	Clock	DNs	LNC	MNC
*L. maderae*	x	x	x	x	x	x	x	x	x
*L. migratoria*	x	x	x	x	x		x	−	−
*D. melanogaster*	−	−	−	x	x	−	x	x ^2^	−
*R. prolixus* ^3^			x		x				x
*A. domesticus* ^3^		x						x	x
*A. aegypti* ^3^								x	x

Notes: x, present; −, not present; no annotation, not clear whether present or not (no statement is provided in papers). Acronyms: CB, central body; AL, antennal lobe; OL, optic lobe; LK, lateral horn; TC, tritocerebrum; SEZ subesophageal zone; DNs, descending neurons; LNC, lateral neurosecretory cells; MNC, median neurosecretory cells. ^1^ The majority of the LK cell bodies are in the protocerebrum and subesophageal zone (SEZ), but processes innervate neuropils in other brain regions. ^2^ In ALK neurons (LNCs), the LK expression is strong in larvae and weak and variable in adults. ^3^ The description of distribution of LK neurons and their processes is not detailed.

**Table 2:** Functional roles of LK signaling in arthropods.

Function	Species	Stage	Reference
Mediate hunger-regulated nociception	*D. melanogaster*	Adult	[129]
Link clock and sleep-regulating neurons	*D. melanogaster*	Adult	[100]
Inhibit postprandial sleep	*D. melanogaster*	Adult	[101]
Starvation-induced sleep suppression	*D. melanogaster*	Adult	[102,103]
Signaling to insulin-producing cells	*D. melanogaster*	Adult	[51,103]
Regulation of feeding and metabolism	*D. melanogaster*	Adult	[51,103]
State-dependent expression of water- and sugar-seeking memories	*D. melanogaster*	Adult	[130]
Modulate food choice	*D. melanogaster*	Adult	[131]
Regulation of defensive post-mating response in females	*D. melanogaster*	Adult	[132]
Induce secretion in renal tubules	Multiple insects	Adult	(see [50,75,133])
Water and ion homeostasis, modulation of desiccation response	*D. melanogaster*	Adult	[87,134]
Myostimulatory action (visceral muscle)	*Multiple insects*	Adult	[9,40]
Larval locomotion	*D. melanogaster*	Larva	[135]
Pre-ecdysis behavior	*D. melanogaster*	Larva	[136]
Tracheal clearance at ecdysis	*D. melanogaster*	Larva	[137]
Regulation of meal size	*D. melanogaster* *A. aegypti* *R. prolixus*	Adult	[113] [138] [110]
Modulation of aversive response to bacteria	*D. melanogaster*	Adult	[139]
Regulation of starvation-induced hyperactivity	*D. melanogaster*	Adult	[140]
Longevity (LK knockdown extends lifespan)	*D. melanogaster*	Adult	[141]
Modulation of sugar taste responses	*D. melanogaster* *A. aegypti* *A. cerana* ^2^	Adult	[141] [138]
Cotransmission in SIFamide neurons	*S. gregaria*	All ^1^	[83]
Cotransmission in PDF clock neurons	*L. maderae*	Adult	[93]
Regulation of digestive enzyme release	*O. arenosella*	Adult	[142]
Labor division in foraging for nectar/pollen in honeybees	*A. cerana*	Adult	[76]
Modulation in stomatogastric ganglion (feeding)	*Cancer borealis* (crab)	Adult	[143]
Regulation of nutrition-dependent fecundity	*R. microplus* (tick)	Adult	[144]

Notes: ^1^ The LK expression in SIFamide neurons is stronger during development, but remains throughout development and adult stage. ^2^ The Asian honeybee *Apis cerana.*

## Data Availability

Data is contained within the article or Supplementary Material.

## Supplementary Materials

The following are available online at https://www.mdpi.com/1422-0067/22/4/1531/s1: Figure S1. The LK precursor and LK peptides in the cockroach *P. americana*. Text File S1. Sequences of LKRs in different species used for the cladogram in Figure 4. (PDF file).
