# Supplementary material for: Leucokinins: Multifunctional Neuropeptides and Hormones in Insects and Other Invertebrates
Source: Int J Mol Sci. 2021 Feb 3;22(4):1531. doi: 10.3390/ijms22041531 (PMC7913504; doi:10.3390/ijms22041531)
Supplement: Supplementary file 1 [file ijms-22-01531-s001.pdf]

Supplementary material file

Leucokinins: multifunctional neuropeptides and hormones in insects

Dick R. Nässel[1]* and Shun-Fan Wu[2]

1. **Supplementary Figure 1:** *Periplaneta americana* leucokinins

2. **Supplementary text file 1:** Sequences of LK receptors used in the phylogenetic tree in Fig. 4

Supplementary Figure 1. *Periplaneta americana* leucokinins

A. >PaSCF24368 Leucokinin precursor (Zeng et al., 2020)

```
MLQMRCRMQLLRAQLLFLAVVFIFHGQSVSGTASSPYHSKPGDAIWEQNSELL
QEDVLSRLLDGPDVLPIDSSRWRSRRDSDSDVDSPPCETTPCPLHNSSSSSSL
SGYSTDEFLSPPDNEPSSPQMKPHIRPTQLKKRVYGRRRDDQQEDSRRQSAPE
GSRIGGRVKRDAGELISETNDELEKREPGFNSWGGKRSPAFSSWGGKRAAPFS
IVGSIRRRAFSSWGGKRSPAFNSWGGKRSNSFSIVSNRHPLFNSWGGGSEPSF
NVLSRDEGPAFRVVSGKRRPSFNSWGGKRDASFSSWGGKRNPAFSILGSSSSE
DPPFSIIGGVDQPAFSIVGGEKRLPAFSSWGGKRDPAFKILGNHEYPAFSVLG
NNYEPAFSIIPSKRDSPFSSWGGKRDPAFSSWGGKREPSFNSWGGKRDPAFSS
WGGKRDDIVSPWQQVTKKGAQFSSWGGKRDSEEGKRSFSSWGGKRGDDSVDGS
KRSFSSWGGKRSPSGEGEHNATSCCDGTQTSVHIDDRQDASTQTSLSSDAYQK
LLEEFDSKIQLENGDGSHGDENGVQGVEEVEMDGENSKGTQTSEPEEEKSENQ
QEEEEEKAENGNQVEEDEAEKEEEENAHTEEEVPEGEVANSHEELPDISSDHE
PATADKSVGTSELSSVIKRTASGNTAGRTRISKALFSPWGGKRSYNKLPPSLL
TVLGSMHKGGASRSNSLLSDLLNKRGNSRLNVLGAKKWGQAPPGAVFSSWGGK
RSDKFSRRNLHKAQSQNVGRQYRRGADFYSWGGK-
```

```
                        EPGFNSWG
                        SPAFSSWG
                          AFSSWG
                        SPAFNSWG
                        RPSFNSWG
                        DASFSSWG
                        LPAFSSWG
                        DSPFSSWG
                        DPAFSSWG
                        EPSFNSWG
                        DPAFSSWG
            DDIVSPWQQVTKKGAQFSSWG
                          SFSSWG
                          SFSSWG
           TASGNTAGRTRISKALFSPWG
       GNSRLNVLGAKKWGQAPPGAVFSSWG
                        GADFYSWG
```

Consensus FXSWG

X = S,N,Y

Only two LKs are identical to ones found in *Leucophaea* (underlined above)

LK7	DPAFSSWG
LK8	GADFYSWG

B. Kinins in *P. americana* (Neupert et al., 2012)

Pea-K-1	949.5	RPSFNSWGa
Pea-K-2	855.4	DASFSSWGa
Pea-K-3	908.4	DPSFNSWGa
Pea-K-4	838.4	GAQFSSWGa
Pea-K-5	864.4	SPAFNSWGa
Pea-K-6	653.4	AFSSWGa
Pea-K-7	865.4	DPAFSSWGa
Pea-K-8	901.4	GADFYSWGa

Figure legends

Supplementary Figure 1. LKs in the cockroach *Periplaneta americana.* **A.** *P. americana* LK precursor (Zeng et al., 2020). The signal peptide is indicated in green, LK peptides in blue, cleavage sites in yellow and putative cleavage sites that appear not to be utilized in red. One LK-like peptide that lack cleavage sites is indicated in black (white text). The LK peptides are listed where those that could be confirmed by mass spectrometry are shown in grey (see also B). Only to LKs are identical to LKs (LK7 and LK8) found in the cockroach *L. maderae* (underlined). Note that two of the LKs exist in two copies in the precursor. Blue lettering indicates dibasic amino acids that appear not to result in peptidase cleavage. **B.** Kinins that were identified by mass spectrometry in *P. americana* (Neupert et al., 2012). Masses are given before sequence. Note that Pea-K-3 (red text) was not found in the precursor in A.

References

Neupert S, Fusca D, Schachtner J, Kloppenburg P, Predel R (2012) Toward a single-cell-based analysis of neuropeptide expression in *Periplaneta americana* antennal lobe neurons. J Comp Neurol 520:694-716

Zeng H, Qin Y, Du E, Wei Q, Li Y, Huang D, Wang G, Veenstra JA, Li S, Li N (2020) Genomics- and Peptidomics-Based Discovery of Conserved and Novel Neuropeptides in the American Cockroach. Journal of Proteome Research ePub

Supplementary Material Text File S1
Sequences of LK receptors used in the phylogenetic tree in Fig. 4

>Drosophila melanogaster LKR
MAMDLIEQESRLEFLPGAEEEAEFERLYAAPAEIVALLSIFYGGISIVAVIGNTL
VIWVVATTRQMRTVTNMYIANLAFADVIIGLFCIPFQFQAALLQSWNLPWFM
CSFCPFVQALSVNVSVFTLTAIAIDRHRAIINPLRARPTKFVSKFIIGGIWMLAL
LFAVPFAIAFRVEELTERFRENNETYNVTRPFCMNKNLSDDQLQSFRYTLVF
VQYLVPFCVISFVYIQMAVRLWGTRAPGNAQDSRDITLLKNKKKVIKMLIIVVII
FGLCWLPLQLYNILYVTIPEINDYHFISIVWFCCDWLAMSNSCYNPFIYGIYNE
KFKREFNKRFAACFCKFKTSMDAHERTFSMHTRASSIRSTYANSSMRIRSNL
FGPARGGVNNGKPGLHMPRVHGSGANSGIYNGSSGQNNNVNGQHHQHQ
SVVTFAATPGVSAPGVGVAMPPWRRNNFKPLHPNVIECEDDVALMELPSTT
PPSEELASGAGVQLALLSRESSSCICEQEFGSQTECDGTCILSEVSRVHLPG
SQAKDKDAGKSLWQPL
>Ramazzottius varieornatus LKR
MDLTSEAWDYSQDFYYLNTSQNHDTFFFTLQPSNGFTSANASTDQGSGEP
PALYEASPAIIAVLAVFYGAISLLALVGNSLVVLCIASSKQMQSVTNFLLANLA
SADILIAVAAVPFQFQAALLQRWVLPEFLCILAPFVQVLSVNVSVFTLTVISIER
YRALLYPFKSRITKSATSYAIAIIWIVAVVCAAPMAASLRVVVMEDDLSGPRLF
CFPSGLSTPVNDSTRAKEEAIEESTRIFRYYVVFLVAIQFAIPLLIISFAYITIALH
LWGTKGPGEGQQDMSLVRIQQRQKVIKMLILVVVLFALSWMPLQTYNFLTAI
IPQINMYSYINIIWFCCNFLAMSNSCQNPFILGFCNEKFRRQLRRKCRWCLGL
RRNNSMAGGGSPNINHHIIRGGASGVLEVGNSPTPLPNGNGVYTDTLYVTM
ALNKGRQFRTASQRSDIRPARAANSLCPSAMNSNEGTSVDDRDYECSVRP
MLHRNHSARSHTKYSIAAEYYD
>Capitella teleta LKR
MFNASDPHFAVSTDVINALNNSVNGSDGNSTDGELLYYVPTGLVVLLSILYG
SISILSVVGNFLVILVIIKNKSMQTVTNFFFANLSVADVMIGIFSIPFQFQAALSQ
RWDLPHILCPVAPFVKELTVNVSVITLTVISIDRYFAVLHPLKARVSRKVAKIV
MSVVWAFSLASAIPVAIVYRVKFTDDKHTNGKKPFCSPTFGTFNDIDLGRVF
HLYVAVVQYFLPLVIICYSYFRIMHRIWLTKAPGSAMDTRDQIMNRNKRKVIK
MLLIVVALFAFCWLPLQTYNLLSIILEKINKYRYINIIWFCSNWLAMSNSCYNPF
IYGLLNEKFKHSFRMLFIKCPCRCARKGLVKNTFCEHSEASEFIRKPTTCPES
TRFQLTNGRIQRYVRVNVVDNTESSFV
>Bombyx mori LKR
MDGSANTSQDDEADWPGNSTLDEYIAQNSTSDVYDTLYDVPTGVIVLLSFL
YGSISVLAVVGNFLVMWVVATSRRMQSVTNCYIANLALADIVIGLFAIPFQFQ
AALLQRWLLPHFMCAFCPFVQALSVNVSVFTLTAIAVDRHRAIITPLSAHTSK
RVAKVIIVFIWLLAFTLAAPMEMSWEVVMEDEIDPGTKLVYKKPFCTASEFGS
NSLAIYRLLLYIFQYVIPLCVITFAYVHMAMKLWGARAPGNAQETRDANHMK
NKKKVIKMLVLVVALFALCWLPLQSYLLLQSFFPSINEYRYINVIFFCFDWLAM
SNSCYNPFIYAIYNEKFKKEFKQRFTFGKKPNRFANDSYEDGQSYRTRILSF
RSTNDRCLYSTRKSINITPDDSLRLSTHSSVQYTNNQSRENGCECTKTEEAQ
ARITARRYANMRMGCRHPNARKCFSKTNETDEMPIGDERVSELYIFPNSNIV
EFRDISYDDKV
>Bactrocera dorsalis LKR
MDEFPVLQIPDDEVLEEEEFERLYSAPIEIVIILSIFYGGISILAIIGNSLVIWVVIT
ARQMRTVTNTYIANLAVADVIIGLFCIPFQFQAALLQRWNLPWFMCAFCPFV
QTLSVNVSVFTLTAIAIDRHRAIINPLRARPSKYISKFILLGIWLSAFIFASPIAVA

FRVEVMHERYREDGVIYNVTRPFCTNVNLSDEQLKAYRYALVIVQYCVPCC
VISFVYVQMAVKLWLTETPGNAEDTRDMALMRNKKKVIKMLIVVVIVFGICWL
PLQLYNVLYVTIPEINEYHFISIIWFCCDWLAMSNSCYNPFIYGIYNEKFKREF
NKRFNLCFCKFRTNNDAHERTLSMHTRATSLRSNFANSSMRIRNNLYAEAA
AGQHKPDIYYTYRYANMRSSRNGSNASAHNNIALPRKTMQTYDYSKTNTEL
HNAAAAAAAANGQSVPQWRRNNFKPLYPDVIECEDDLDTLMHSTPTSEEP
NSSSAGSNKVNMSAGPGFIFKTTGKR
>Anopheles gambiae LKR
MQATDVTAYHTAYNYTLNQSDVRIVLEDENLYKVPIGLLVLLSLFYGTISILAVI
GNSLVIWIVLTTKQMQTITNMFIANLALADVTIGVFAIPFQFQAALLQRWNLPE
FMCPFCPFVQLISVNVSVFTLTAIAVDRHRAIINPLRARTSKNISKFVISTIWML
SFALAAPILFALRVRPVSYIALGGMNETYTNITVPFCKVVNFEHGEIQLYRYVL
VLVQYFVPLFVISFVYIQMALRLWGSKTPGNAQDSRDITMLKNKKKVIKMLIIV
VALFGVCWFPLQLYNILHVTWPEINEYRFINIIWFVCDWLAMSNSCYNPFIYG
IYNEKFKREFRKRYPFKRDQTYNHNHESDKTSSIFTRVSSVRSTYATSSIRN
KLSTNRYSGSKQLPAQFKFPPAGHHFPHHPVGGGHLHELSFGGPRKGAGE
GLSVTSGTVTTNFGQNRPPSWEKRMEEPRQNEHEKLIVSSAASLDQQLAS
RQLTEDSVGPSDGFGSRIGAARRNGSARESTSTVRMALNHPHPDSGGESG
GDGEPNRAHSNGTADAKGGHLYCNDLEQLGPYFD
>Cryptotermes secundus LKR
MRTARSSETPIDICFQKKAMSESKFSLNGSLDRYSYSIANYSGDHGRAPSSQ
FEMDVSPSFNDSESGNSSMEDYLNSGSLLPNETYDSLFEVPTGVTALLSLF
YGSISVMAVVGNSLVMWIVATSRRMQNVTNCFIANLALADIVIGLFAIPFQFQ
AALLQRWNLPHFMCAFCPFVQVLSVNVSIFTLTAIAVDRHRAILNPLSASPSK
LCAKLVIAGIWVISGGLAAPMAVALRVTMVDSPHGHQKPFCHNVKLSEEAML
SYRVILVVLQYLTPLCIISCVYIRMALALWGSKAPGNAQSSRDATLMKNKMKV
IKMLVIVVALFALCWLPLQTYNVLHNIFPEINGYRYINIIWFCCDWLAMSNSCY
NPFIYGIYNEKFKREFQLRFGLCSRRCSAAAPGTSLTRDLSEMEKLSSRYDN
SFRYHPTCPPPPPPPQGRRDAYRTLLTQNHRTRPICADEKY
>Rhipicephalus microplus LKR
MTSLPGMTLDPSAPPPLLLDSSYVSPDYGNLSLLSSLPAANISSNKLYQVPV
GFIVLLSIFYGIISLVAVAGNFMVMWIVATSRRMQTVTNFFIANLAVADIIIGLFS
IPFQFQAALLQRWVLPEFMCAFCPFVQVLSVNVSIFTLTAIALDRYRAVMSPL
KARTTKLRAKFIICGIWTLAVAAALPCALALRVETQVESHALNLTKPFCHEVGI
SRKAWRIYNHVLVCLQYFFPLLTICFVYARMGLKLKESKSPGNAQGARDAGI
LKNKKKVIKMLFVIVALFAFCWLPYQLYNILREVFPKIDKYKYINIIWFCTHWLA
MSNSCYNPFIYAIYNERFKREFATRCTCGGHRYKSPKSRFASYEQEDNSTII
VSMRHSFRLSFKNSAPLKASTQV
>Aedes aegypti LKR
MRAVDGIAFHYANNNTLNGSDVEIVKEQDALYDVPVGLVVLLSIFYGTISIIAVI
GNSLVIWIVLTTKQMQTITNMFIANLALADVTIAVFAIPFQFQAAVLQRWNLPE
FMCPFCPFVQLLSVNVSVFTLTAIAVDRHRAIINPLRARASKNISKFVISAIWM
MSFALAAPTLFALRVVPVSIVSLGETNETYINMTKPFCQVVNFEESEMLLYRY
ILTLVQYFVPLCVISFVYIQMALRLWGSKTPGNAQDSRDMTMLKNKKKVIKM
LIIVVALFGICWFPLQLYNILHVTWSEVNEYRYINIIWFVCDWLAMSNSCYNPF
IYGIYNEKFKREFHKRYPFRGRNQSYHQEQLTDKTLSMFTRVSSIRSNYATS
SIRNKLYTGPIGGGSGNGGTHVGSGYSSNAFYQNQNSHHQQSYKSPNTNS
VAGYQRNSTTDRNSSRKTAAGAPWDPKCCPCRQNSTRTSTAAASACPYR
MPLPAVASDGDSGSEGGPCNSAGGGQSPMINNDERQLLGADDNYGSAAQ

KLEVISLDHPHPDSADDENGVAETPHSRTANGQEQDERLQLTSFISSGNGR
HERFHFHINNL
>Aplysia californica LKR
MDGLGAVAEQAAFIIVEDVSRSNNGDNSTSSFSSSSSLSSSSDSIMADLLGT
NTTASSLPGNTTDSEPYDVPTGLMVLLAFLYGSISLMAVIGNGLVILVIVKNRR
MHTVTNIFIANLAVADVIIGIFSIPFQFQAAILQRWVLANFLCSLAPFVQLISVNV
SIFTLTVIAVDRYIAVIHPFKAGCSKRSAAIIISVIWTVAVGS
>Lymnaea stagnalis LKR
MSQIESMSEQAAVIFIEQANQDLDNVSGNDVSSFFYNETTTLFPGSNESFVM
PYDVPTGLICLLAFLYGSISLLAVIGNGLVILVIVKNRRMHTVTNIFIPNLAVSDV
IIGLFSIPFQFQAALLQRWVLANFMSSLPPFVQVVTVNLTIFTLRVIAVDRYIAV
IHPFKAGCSKKRAAIIISIIWAVGIGAALPVPLFYWVEDLTENNIVIPRCDWHAP
DNWLDFHLYYNTLLVCFQYLLPLVIITYCYCRIAWHIWGSRRPGAHVTTEDV
RGRNKRKVVKMMIIVVCLFVLCWLPLQMYNLLHNINPLINHYHYINIIWFSSN
WLAMSNSCYNPFIYGLLNEKFKREFHQLFVMCPCWKARVDYYTEYFSEDA
NICRRANTNGHCPANRHGAVGTTSTETTRKSMLSRSRCKGTRRRRQTYDE
RRETSS
>Musca domestica LKR
MDEYGITRFKENEMLDDEEYEVLYIAPVEIVVLLSVFYGTISLVAIVGNSLVIW
VVATTRQMRTVTNMYIANLAFADVIIGLFCIPFQFQAALLQRWTLPWFMCGF
CPFVQAVSVNVSVFTLTAIAIDRHRAILNPLRARPTKFASKFIIATIWLASVTFA
TPCAIAFRVELQHERRKDTINNTIYNVTTPFCNNVNLSDSQLQTYRYSLVFVQ
YLVPFCVISFVYIQMAIRLWGTRAPGNAQDSRDITLMRNKKKVIKMLIIVVIVF
GVCWLPLQLYNILYVTIPEVNEYQFIGIVWFCCDWLAMSNSCYNPFIYGIYNE
KFKREFNKRFQTFFCHFHTTGDIHERTMSMHTRASSLRSTYGTSRLRNQNR
TSSIYNRQTSSERDSKRGEAQQLCNFITYNVNSNGEEILLSGLTPTNGIELKT
KEIESVRPNSGWQWQRNNFKPLHPELLECDSSEEQVLVGDKHPPHSEALR
EFPVNGYSGNKANNEETDILRAHHQQTIM
>Penaeus vannamei LKR
MEEPASYVMTQVLEDPYKSLLENLSVQYPHIDWWGPFNITSYNLTDFPFLLD
DDDGAGGGGRRGTGGGLGYMESAGEASNATGGAALLYEVPTSLVVLLSFF
YGSISLVAVVGNALVMWVVATSKKMHTVTNYFIANLALADIIIGLFAIPFQFQA
ALLQRWNLPHFMCSFCPFFQTVSVNVSIFTLTAIAVDRYRAIVHPFTTRPSKL
RSKVVIASIWLFSTTLAIPNAIALRVVRVPDEATGRDKPYCAAVNIDSVVMWT
YSHVMVGLQYFLPLGIISFAYIRMGWELWGAQXPGNAEDARDAHVLRNKKK
VIKMLSMVVILFAVCWAPLQTYHVLQEIYPAINRYRYINIIWFCCHWLAMSNS
CCNPFIYAIYNEKFKREFRLKFRCCFRHLDSLEALDIEKSKCHFPVGGPEVHL
HKATLLSSPPDLRRNGGSSASPYVIRPVHEHERSHEVHVCVPLKAQARLHP
DRHCPNKCGDTKIKRLCCAADPGLNSCSWGKALVARVMAMMYGYYTRTM
NSAPKDTLLTKGTFFFGFPWCHVARVENGLGLWRVSTWRLAGTYQGRVG
WVRVEVLGIF
>Tetranychus urticae (tetur02g07280)
MDITRYKFGITNSSSLVSASSYLIKSILTNVSPSASPLSLTSLPSSPLSSIVVLP
PSSSSSTSPPSPLPPSEASLSGDESIDNSEASSEGIYQIPPLMIVFLTSIYLLVS
LCAVIGNSMVLWIVIKSKRMRNVTNFFIANLAVADLVIASLAVPFQFPAALLQR
WVLPHFMCSLCPTAQVVSVNVSIFTLVAISLDRHQAVTRPLATRMYKKSALFI
IAFIWLISLLLATPTFVAWEVRYVWSTESSNYTEPFCDTSSVSSDFWRHYNH
FLVALQYFIPLFVISFAYIHMAVILSEVDATTAKRNDYMRALQNKRRVIKMLFIV
VALFAICWLPFQLYNILQEIYPSINEYKYINVIWFSCHWLSMSNSCYNPFIYAIY

GERFKAEFVARFRICRVLRKCMVTDDKSTNGLTYSFTGRKFADTSLYKKSIG
SSAAAE*
>Tetranychus urticae (tetur03g03420)
MFDNFTFSVNDEINFESYNKTLSLFSEELLYSFPLGVVVLLVLAYGIVSLTAVI
GNTIVLCCVVGSVRLRTVTNMFIANLATADILIGALSIPFQFQAALLQKWILPPF
MCAFCPFIQIVSVNVSIYTLVAIAADRYLVVFAPLHCRINKYRARMIIIFIWAWS
VIAAIPALIALRVRLVPDLESAVDSHDDKWLRSLVTGNLTIPTRPSCDNIGMSE
SIWRGYNQFLVVIQYFLPLGIITFAYTRMASKLHGQEKSGSSGLISLDNNKSS
HQGDSVFRNSQLVTSYHLTTADAEYIVTHKRKVMKMLIVVVALFALCWLPLQ
TYNLLKDTIPEINTFRYINVIWFCCHWLAMSNSSVNPFIYAIYKVTIDH*
>Drosophila melanogaster NPFR
MIISMNQTEPAQLADGEHLSGYASSSNSVRYLDDRHPLDYLDLGTVHALNTT
AINTSDLNETGSRPLDPVLIDRFLSNRAVDSPWYHMLISMYGVLIVFGALGNT
LVVIAVIRKPIMRTARNLFILNLAISDLLLCLVTMPLTLMEILSKYWPYGSCSILC
KTIAMLQALCIFVSTISITAIAFDRYQVIVYPTRDSLQFVGAVTILAGIWALALLL
ASPLFVYKELINTDTPALLQQIGLQDTIPYCIEDWPSRNGRFYYSIFSLCVQYL
VPILIVSVAYFGIYNKLKSRITVVAVQASSAQRKVERGRRMKRTNCLLISIAIIF
GVSWLPLNFFNLYADMERSPVTQSMLVRYAICHMIGMSSACSNPLLYGWLN
DNFRKEFQELLCRCSDTNVALNGHTTGCNVQAAARRRRKLGAELSKGELKL
LGPGGAQSGTAGGEGGLAATDFMTGHHEGGLRSAITESVALTDHNPVPSE
VTKLMPR
